# Pigment Epithelium-Derived Factor Inhibits Cell Motility and p-ERK1/2 Signaling in Intrahepatic Cholangiocarcinoma Cell Lines

**DOI:** 10.3390/biology14020155

**Published:** 2025-02-03

**Authors:** Veronica Porreca, Eleonora Corbella, Biagio Palmisano, Marco Peres, Pietro Angelone, Cristina Barbagallo, Michele Stella, Giuseppina Mignogna, Gianluca Mennini, Fabio Melandro, Massimo Rossi, Marco Ragusa, Alessandro Corsi, Mara Riminucci, Bruno Maras, Carmine Mancone

**Affiliations:** 1 Department of Molecular Medicine, Sapienza University of Rome, 00161 Rome, Italy; veronica.porreca@uniroma1.it (V.P.); corbella.2016275@studenti.uniroma1.it (E.C.); biagio.palmisano@uniroma1.it (B.P.); peres.1461165@studenti.uniroma1.it (M.P.); angelone.1881277@studenti.uniroma1.it (P.A.); alessandro.corsi@uniroma1.it (A.C.); mara.riminucci@uniroma1.it (M.R.); 2 Department of Biomedical and Biotechnological Sciences-Section of Biology and Genetics, University of Catania, 95123 Catania, Italy; cbarbagallo@unict.it (C.B.); michele.stella@unict.it (M.S.); mragusa@unict.it (M.R.); 3 Department of Biochemical Science, Sapienza University of Rome, Piazzale Aldo Moro 5, 00185 Rome, Italy; pina.mignogna@uniroma1.it (G.M.); bruno.maras@uniroma1.it (B.M.); 4 General Surgery and Organ Transplantation Unit, Department of General Surgery and Surgical Specialties P. Stefanini, Sapienza University of Rome, Viale del Policlinico 155, 00161 Rome, Italy; gianluca.mennini@uniroma1.it (G.M.); fabio.melandro@uniroma1.it (F.M.); massimo.rossi@uniroma1.it (M.R.)

**Keywords:** intrahepatic cholangiocarcinoma, tumor microenvironment, PEDF, cell migration, extracellular matrix

## Abstract

Pigment epithelium-derived factor (PEDF) is a multifunctional soluble glycoprotein, primarily known for its potent anti-angiogenic properties. In recent years, its ability to counteract cell proliferation and motility has generated interest in PEDF as a potential tumor suppressor. In the intrahepatic Cholangiocarcinoma (iCCA), PEDF, Thrombospondin 1 (THBS1), and Thrombospondin 2 (THBS2) are expressed and released into the tumor microenvironment (TME), where they promote lymphangiogenesis at the expense of the neoangiogenic program, aiding the dissemination of cancer cells via lymphatic vessels. Recently, we demonstrated that THBS1 and THBS2 directly affect iCCA cells, exacerbating their malignant behavior, while the direct role of PEDF remains to be elucidated. In this study, through a cell-based assay and molecular analysis, we investigate the direct function of PEDF on two well-established iCCA cell lines. Our results show that PEDF affects cancer cell motility in a paracrine manner, reducing their migratory and invasive capabilities. Notably, our data suggest that the PEDF-induced inhibition of motility in iCCA cells occurs through the MAPK/ERK signaling pathway, as indicated by the reduced phosphorylation of ERK1/2. Overall, this study provides the first evidence of PEDF acting as a tumor suppressor in iCCA.

## Simple Summary

Intrahepatic cholangiocarcinoma (iCCA) is a highly aggressive primary biliary epithelial cancer. The iCCA microenvironment significantly contributes to the malignancy of tumor cells by releasing soluble mediators from non-cancer cells. Among these mediators, pigment epithelium-derived factor (PEDF), Thrombospondin 1 (THBS1) and 2 (THBS2) are highly expressed in iCCA tissue, where they act as inhibitors of angiogenesis and promoters of lymphangiogenesis, thereby facilitating tumor cells dissemination. Although THBS1 and THBS2 directly affect iCCA tumor cells by enhancing their malignant traits, the direct role of PEDF in iCCA cells remains unexplored. This will be the focus of the proposed study. PEDF, primarily known for its anti-angiogenic properties, has attracted interest due to its potential anti-metastatic activity in various cancer types. Our results show that PEDF affects iCCA cell motility by reducing their migratory and invasive capabilities. Specifically, we demonstrate that PEDF decreased ERK1/2 phosphorylation, suggesting that the inhibition of motility induced by PEDF occurs through the MAPK/ERK signaling pathway. Future studies focused on identifying the PEDF receptor will facilitate the development of therapeutic strategies designed to counteract the migratory and invasive capacities of iCCA cells, and thereby reducing their aggressiveness.

## 1. Introduction

Pigment epithelium-derived factor (PEDF) is a soluble monomeric glycoprotein that plays a crucial role in various biological processes, both physiological and pathological [1,2]. Initially identified as a neurotrophic factor secreted by retinal pigment epithelial cells, PEDF is now known to be expressed in a wide range of tissues, notably the brain, liver, and plasma, where it is present at concentrations exceeding 500 ng/mL [3,4,5]. Encoded by the *SERPINF1* gene, PEDF belongs to the subgroup of non-inhibitory serpins, which have lost protease inhibitor activity and instead promote functions such as neurogenesis, inflammation, stem cell renewal, cell proliferation and survival [2,6,7]. However, PEDF is more widely known for its potent ability to inhibit angiogenesis, attracting considerable attention to its potential role in cancer [8,9,10,11].

Although it has been recently reported as a promoting factor of ovarian cancer dissemination [12], PEDF is known for its indirect anticancer activity of inducing apoptosis in endothelial cells (ECs), inhibiting their migration and reducing the release of pro-angiogenic factors within the tumor microenvironment (TME) [9,10]. Furthermore, PEDF can directly affect tumor cells by promoting apoptosis, driving the differentiation towards a less-malignant phenotype and decreasing cancer cell migration and invasion [9,10]. Despite these well-documented anticancer and antimetastatic activities, which have been demonstrated in various in vitro and in vivo models, the reduced levels of PEDF found in many solid tumors seem to overwhelm its potential therapeutic effects. In a glioma, the absence of a PEDF expression is correlated to tumor progression [13]. In fact, the expression of PEDF affects the glioma TME by downregulating the production of matrix metalloproteinase-9 (MMP9), the vascular endothelial growth factor (VEGF), and the basic fibroblast growth factor (bFGF), leading to the inhibition of cancer cell invasiveness and promoting the apoptosis [14]. A reduced PEDF expression was also observed during the progression of human breast cancer [15]. The intratumoral expression of PEDF inhibits breast cancer cell migration and invasion by downregulating fibronectin, and, in turn, reduces the metalloproteinase-2 (MMP2)/MMP9 in the TME via p-ERK and p-AKT signaling pathways [16]. In pancreatic cancer tissues, decreased PEDF levels are associated with an increased tumor angiogenesis, the development of a liver metastasis, and a poor prognosis [17]. Moreover, it has been observed that the extracellular PEDF blocked Wnt3a-directed induction of an autophagy in pancreatic intraepithelial neoplasms, thus exerting a preventing role in the autophagy-induced pancreatic carcinogenesis [18].

Recent data indicate that PEDF is highly expressed in primary liver cancers, such as the hepatocellular carcinoma (HCC) and the intrahepatic cholangiocarcinoma (iCCA) [4,19,20]. In HCC, the upregulation of PEDF is not associated with patient prognosis due to the balancing effects of its intracellular and extracellular activities. While extracellular PEDF inhibits angiogenesis, the intracellular PEDF expression contributes to the accumulation of free fatty acids, which promote HCC cell growth [19]. Recently, for the first time, we reported on the expression of PEDF in iCCA by unveiling its overexpression in cancer specimens, compared with the adjacent non-cancerous tissues (NCT) [20]. Specifically, we demonstrated that PEDF was mainly expressed by the cancer-associated fibroblasts (CAFs), indicating that the intracellular autocrine effects of PEDF were negligible for the malignant cholangiocytes. Thus, the CAFs are the pivotal players in orchestrating the paracrine action of PEDF in modulating the iCCA tumor stroma, particularly for the angiogenic and lymphangiogenic program. In fact, along with two other angiogenesis inhibitors, thrombospondin 1 (THBS1) and 2 (THBS2), PEDF displayed antiangiogenic activity in iCCA TME. In mouse xenograft experiments with the iCCA cell line CCLP1, the simultaneous depletion of PEDF, THBS1, and THBS2 in the iCCA TME reduced tumor growth and the extent of the tumor dissemination in local lymph nodes by reducing the neo-lymphangiogenesis arising from the trans-differentiation of vascular endothelial cells towards a lymphatic phenotype. These observations suggest that these angiogenesis inhibitors may play both direct (promoting cell malignancy) and indirect (anti-angiogenetic and lymphangiogenic) roles in driving iCCA progression. Indeed, we recently demonstrated that both THBS1 and THBS2 enhance the malignant phenotype of iCCA cells by increasing cell proliferation, motility, invasion, and adhesion [21]. Conversely, the specific role of PEDF on influencing the iCCA cell behavior remains to be clarified.

Here, despite its indirect tumor-promoting role in inducing iCCA-associated lymphangiogenesis, we sought to verify whether PEDF in iCCA exerted antitumor effects on cancer cells, as observed in glioma, breast, and pancreatic cancers. We present new data, indicating that PEDF inhibits the motility of iCCA cell lines without affecting their proliferation.

## 2. Materials and Methods

### 2.1. Cell Culture

Human iCCA cell lines, CCLP-1 and HuCCT-1, used in this study, were kindly provided by Prof. D. Alvaro (Sapienza University of Rome) and F. Marra (University of Florence), respectively. CCLP1 are mucin-negative mesenchymal cells, while HuCCT-1 are mucin-positive epithelial cells [22]. The CCLP1 cell line was isolated from a 48-years-old Caucasian female and carried *CTNNB1* and *TP53* mutations, while the HuCCT-1 cell line was isolated from a 56-years-old Japanese male and carried *KRAS*, *MSH6* and *TP53* mutations, as reported in Cellosaurus database (https://www.cellosaurus.org/index.html, accessed on 10 January 2025). Both iCCA cell lines were maintained in a RPMI 1640 medium containing 10% fetal bovine serum (FBS) and 2 mM L-glutamine (Lonza, Basel, Switzerland). The medium was supplemented with 1% penicillin-streptomycin (Gibco/BRL; Life Technologies). Cells were cultured at 37 °C in a humid atmosphere of 5% CO_2_ in air. CCLP1 and HuCCT-1 were treated with human recombinant PEDF (rhPEDF), purchased by R&D Systems Europe Ltd. (Abingdon, Oxfordshire, UK). Cellular functional assays and molecular analysis were performed at a dose of 1000 ng/mL of rhPEDF versus control. This concentration was chosen because it is known to inhibit cell proliferation and migration [23].

### 2.2. RNA Isolation and Real-Time qPCR

Total RNA was extracted from CCLP1 and HuCCT-1 cells using the TriZol Reagent (Thermo Fisher Scientific, Waltham, MA, USA), according to the manufacturer’s instructions. The quantification and purity of total RNAs were assessed using a NanoDrop One spectrophotometer (Thermo Fisher Scientific) and a Qubit fluorescence quantification system (Thermo Fisher Scientific). Total RNA was stored at −80 °C until use. To analyze the expression levels of the PEDF transcript in iCCA cell lines, specific PCR primers were designed using the online tool PrimerBlast (http://www.ncbi.nlm.nih.gov/tools/primer-blast, accessed on 18 June 2024). PEDF was amplified using the following primers: PEDF Forward 5′-*GGCTGTTTTACGCTATGGCTTG*-3′; PEDF Reverse 5′-*TCTGGGTCACTTTCAGGGGC*-3′. To normalize the expression data, the GAPDH gene was used as an internal reference, and the PCR primers were reported as follows: GAPDH Forward 5′-*TGCACCACCAACTGCTTAGC*-3′; GAPDH Reverse 5′-*GGCATGGACTGTGGTCATGAG*-3′. Real-time PCR was carried out using the Power SYBR Green RNA-to-CT™ 1-Step Kit (Thermo Fisher Scientific) with 20 ng of total RNA per reaction, according to the manufacturer’s instructions. The experiments were conducted on a 7900HT Fast Real-Time PCR System (Thermo Fisher Scientific). RNA expression levels of PEDF in iCCA cell lines were compared to a commercial human ovary RNA (Ambion^®^), used as a positive control of high expression levels of the transcript [24]. Results were analyzed through SDS RQ Manager 1.2 software (Thermo Fisher Scientific) and the data are shown as 2^−ΔCt^ to estimate the PEDF expression level in iCCA cells, in comparison with the positive control.

### 2.3. Cell Proliferation Assay

Cells were counted manually using a hemocytometer and seed in 96-well plates for achieving a cell density of 5 × 10^3^ cells per well. A proliferative assay was then conducted on iCCA cell lines treated with or without rhPEDF (1000 ng/mL), using the colorimetric 3-(4,5)dimethylthiazol-2-yl)-2,5-diphenyltetrazolium bromide (MTT) assay kit (CyQUANT™ MTT Cell Viability Assay V13154, Invitrogen, Thermo Fisher Scientific, Waltham, MA, USA), as previously reported [21].

### 2.4. Adhesion Assay

To assess the involvement of rhPEDF in cell adhesion, CCLP1 and HuCCT-1 cells were seeded into Matrigel-coated wells (Corning^®^ Matrigel^®^ Growth Factor Reduced (GFR) Basement Membrane Matrix, Corning, New York, USA). For the preparation of the assay, a thin layer of Matrigel (5 μg/well) was coated on a 24-well plate in cold conditions and incubated for 1 h at 37° to promote its solidification. Once solidified, tumor cells were seeded into a 24-well plate at a density of 2.5 × 10^4^ cells per well. Subsequently, cells were treated with 1000 ng/mL of rhPEDF and incubated at 37 °C in a humidified atmosphere of 5% CO_2_. After 45 min of incubation, the plate was shaken at 500 rpm for 15 s and the non-adherent cells were washed off with PBS 1X. The remaining adherent cells were fixed in 100% methanol for 15 min and stained with 0.1% crystal violet (Santa Cruz Biotechnology, Dallas, TX, USA, sc-207460). Adherent cells were subsequently imaged by capturing four optical fields using a light microscope with 4× magnification and employing the ZEN 2.0 software (Carl Zeiss, Oberkochen, Germany). The number of adherent cells was quantified using ImageJ 1.54 software through direct cell counting. Results are presented as the mean of the cell counts per optical field of biological triplicates.

### 2.5. Transwell Assays

Cell migration and invasion of iCCA cell lines were performed by transwell assays, using 24-well cell culture inserts (ThinCert™ 8 µm pore size, Greiner bio-one, Vilvoorde, Belgium). The main difference between the two assays lies in the use of Matrigel, which was employed exclusively in the invasion experiments. For the preparation of the invasion assay, cell culture inserts were coated with Matrigel (0.5 μg/μL) in cold conditions and incubated at 37° for 1 h to allow Matrigel to solidify. In both of the transwell assays, a total of 2 × 10^5^ cells per well were seeded on the apical side of the chamber in a low-FBS medium (RPMI 1640 medium supplemented with 0.2% FBS) to provide essential nutrients while minimizing the serum’s concentration. RPMI supplemented with 10% FBS were added to the basal compartment, either with or without 1000 ng/mL of rhPEDF. After 48 h, the remaining cells on the apical side of the chamber were gently scraped off using wet cotton swabs. Subsequently, migrated or invaded cells were fixed in methanol 100% for 15 min and stained with crystal violet 0.1% for 15 min at room temperature. Migrated or invaded cell images were acquired by capturing six optical fields using a light microscope with 20× magnification and employing the ZEN 2.0 software (Carl Zeiss, Oberkochen, Germany) and quantified using ImageJ 1.54 software through direct cell counting. Data are presented as the mean of cell counts per optical field of three independent experiments.

### 2.6. Western Blot Analysis

CCLP1 and HuCCT-1 cells, treated with or without 1000 ng/mL of rhPEDF for 24 h, were collected and total proteins were extracted using Cell Extraction Buffer (#FNN0011, Thermo Fisher Scientific) supplemented with a protease inhibitor cocktail according to the manufacturer’s instructions. Protein concentrations in each lysate were measured using the BCA method. Western Blot analysis was performed as previously reported [21]. Blots were visualized using LuminataTM Western-HRP Substrate (Millipore Corporation, Billerica, MA 01821 U.S.A) and imaged with the ChemiDocTM Touch Imaging System (Bio-Rad Laboratories, Hercules, CA, USA). Band densities were quantified by ImageLab software version 5.1.2 (Bio-Rad) by considering the background subtracted volumes. The relative level of β-catenin was normalized to the reference protein GAPDH. Conversely, the effect of rhPEDF on the phosphorylation rate of AKT and ERK1/2 has been reported as the ratio of the band density of the phosphorylated form to the total form. SeeBlue™ Plus2 Pre-stained Protein Standard (#LC5925, Thermo Fisher Scientific) and Prestained Protein Ladder—245 kDa (#PL0245, Geneaid) were used as molecular size markers. The following primary antibodie dilutions were used: anti-PEDF (1:500, Abcam, #14993), anti-PARP (1:1000, Cell Signaling, #9532), anti-PCNA (1:1000, Cell Signaling, #13110), anti-AKT (1:1000, Cell Signaling, # 4691), anti-pAKT (1:1000, Cell Signaling, # 4060), anti-ERK1/2 (1:1000, Cell Signaling, # 4695), anti-pERK1/2 (1:1000, Cell Signaling, # 9101), β-catenin (1:500, BD Biosciences, #610153), anti-actin (1:1000, Santa Cruz Biotechnology, #sc-1615), and anti-GAPDH (1:1000, Santa Cruz Biotechnology, #sc20357). Additional details on the validation and specificity of the antibodies used are reported in Table S1.

### 2.7. Statistical Analysis

Statistical analysis was performed using GraphPad Prism version 8 software (GraphPad Software, Boston, Massachusetts USA). Data normality was initially assessed with the Shapiro-Wilk test. Depending on the results, either the parametric or the non-parametric Student’s *t*-test was performed for comparison between the two groups. *T*-test data are presented as mean ± standard deviation (SD) and were obtained from at least three independent experiments; statistical significance was set at *p* < 0.05.

## 3. Results

### 3.1. rhPEDF Does Not Affect CCLP1 and HuCCT-1 Cell Growth In Vitro

**Figure 1:**
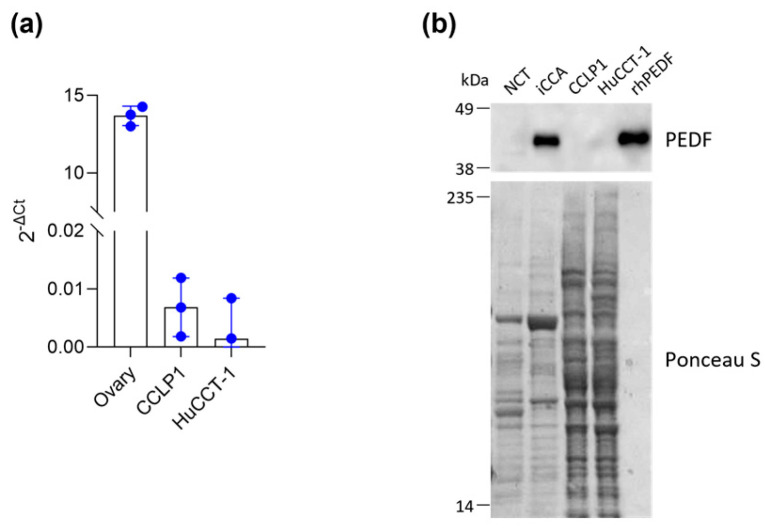
CCLP1 and HuCCT-1 cell lines do not express PEDF. (**a**) PEDF mRNA expression in iCCA cell lines, (CCLP1 and HuCCT-1) was analyzed using Real-Time PCR. Commercial human ovary RNA was used as a positive control. The bar graph illustrates the mean 2^−ΔCt^ of PEDF mRNA normalized with respect to the expression of GAPDH. (**b**) Western blot analysis for PEDF in total tissue extracts from non-cancerous (NCT) and cancerous tissues (iCCA) of a representative iCCA patient as well as total cell extracts from CCLP1 and HuCCT-1 cell lines. For each gel lane, 10 μg of protein were loaded. A total of 10 ng of rhPEDF was used as a positive control. Ponceau S staining of the nitrocellulose membrane is shown to confirm equal protein loading.

To evaluate the robustness of our in vitro cell models, we first examined PEDF expression levels on both epithelial (HuCCT-1) and mesenchymal (CCLP1) iCCA cell lines (Figure 1). PEDF was weakly expressed at the mRNA levels (Figure 1a), as shown by the comparison with the ovary tissue, where it is abundantly expressed [24], and the protein was not detected in both the iCCA cell lines (Figure 1b). These findings confirm that the effects of PEDF are exclusively due to its release by fibroblast cells within the TME. To replicate the CAFs-mediated paracrine action of PEDF on tumor cells, we utilized the recombinant human form of PEDF (rhPEDF) that shows the same migration pattern on SDS-PAGE as PEDF detected in the iCCA tissues (Figure 1b).

**Figure 2:**
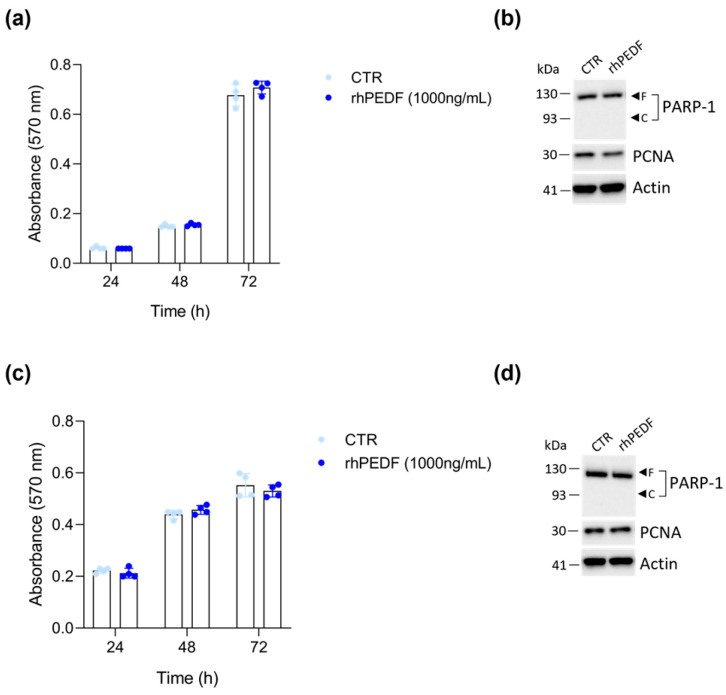
In vitro cell growth is not influenced by rhPEDF treatment. The time course of cell growth for CCLP1 (**a**) and HuCCT-1 (**c**) cell lines was assessed by MTT cell viability assay at the indicated time points in the presence of 1000 ng/mL of rhPEDF; CTR represents untreated cells. Western blot analysis for PCNA and PARP-1 on whole cell extracts of CCLP1 (**b**) and HuCCT-1 (**d**) cells, cultured in the presence of 1000 ng/mL of rhPEDF for 24 h, was performed. Arrows indicate the expected molecular weights for the PARP-1 full-length “F” and cleaved “C” forms; actin was used as a loading control.

CCLP1 and HuCCT-1 cells were subjected to a 1000 ng/mL dose of rhPEDF to assess its effects on cell proliferation. The time course of CCLP1 growth, assessed by MTT cell viability assay, revealed no effect of rhPEDF on cell proliferation (Figure 2a). Additionally, no changes in the proliferating cell nuclear antigen (PCNA) expression or cleavage of the Poly(ADP-ribose) polymerase 1 (PARP-1) by caspases were detected following rhPEDF treatment (Figure 2b). Similar results were obtained when HuCCT-1 cells were exposed to the same 1000 ng/mL dose of PEDF (Figure 2c,d).

### 3.2. rhPEDF Inhibits Cell Adhesion of the HuCCT-1 Cell Line

**Figure 3:**
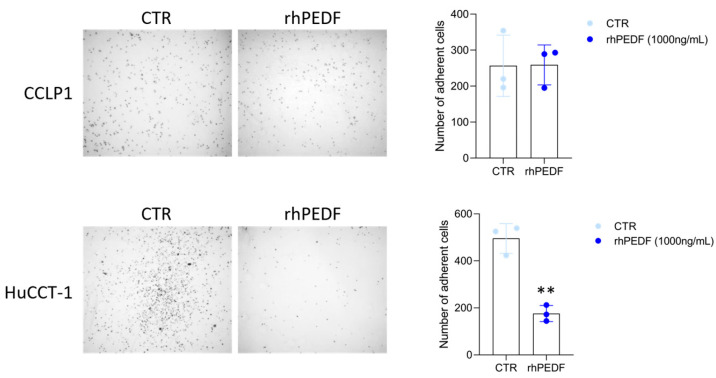
rhPEDF inhibits the cellular adhesive properties of the epithelial HuCCT-1 cell line. Adhesion of CCLP1 and HuCCT-1 cells in response to treatment with (rhPEDF) or without (CTR) 1000 ng/mL of rhPEDF for 45 min. The left panels show representative images (4× magnification) of CCLP1 and HuCCT-1 adherent cells. The bar graphs represent the mean value ± SD of cell counts per four optical fields of three independent experiments ** *p* < 0.01 (*t*-test).

Cancer cells exhibit an adhesion strength to the extracellular matrix (ECM) up to three times stronger than in normal cells [25]. This enhanced interaction with the ECM drives cancer cells toward a more malignant phenotype by conferring resistance to mechanical stresses and promoting cell survival and metastasis [26]. Therefore, we aimed to determine whether rhPEDF could alter the adhesive properties of CCLP1 and HuCCT-1 cells. We observed a statistically significant decrease in the attachment of HuCCT-1 cells upon rhPEDF treatment, while no changes were observed in the adhesion of the CCLP1 cells (Figure 3).

### 3.3. rhPEDF Inhibits CCLP1 and HuCCT-1 Cell Migration, Invasion and p-ERK1/2 Signaling

**Figure 4:**
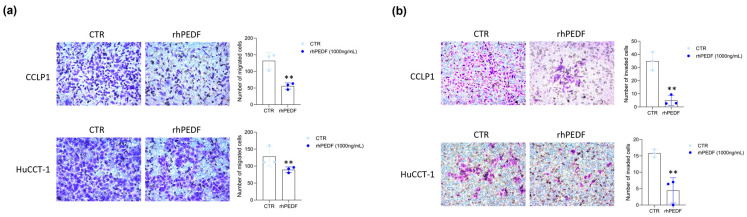
rhPEDF treatment restrains cell migration and invasion in iCCA cell lines. Transwell migration (**a**) and invasion (**b**) assay of CCLP1 and HuCCT-1 cells in response to treatment with (rhPEDF) or without (CTR) 1000 ng/mL of rhPEDF for 48 h. The left panels show representative images (20× magnification) of each experimental group. The bar graphs represent the mean value ± SD from independent experiments performed in triplicate; ** *p* < 0.01 (*t*-test).

Since PEDF primarily exerts its antitumor effect by reducing cancer cell motility, we examined the chemo-attractant effect of rhPEDF on the migration and invasiveness of the CCLP1 and HuCCT-1 cells using transwell migration and invasion assays. Treatment with 1000 ng/mL dose of rhPEDF significantly reduced the migration capacity of both iCCA cell lines (Figure 4a). Similarly, the number of invasive cells following the rhPEDF treatment was significantly lower compared to the untreated cells (Figure 4b).

**Figure 5:**
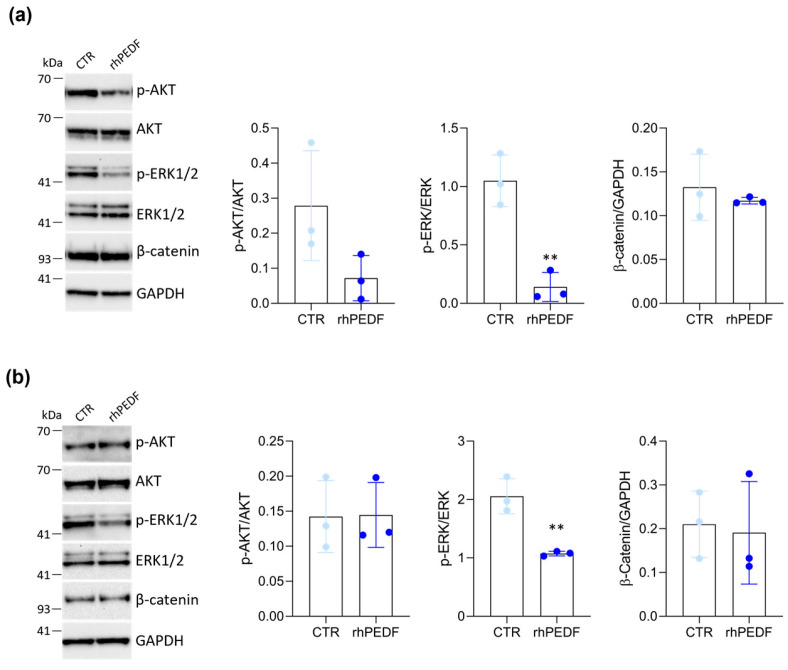
rhPEDF downregulates ERK1/2 phosphorylation. Western blot analysis for the indicated proteins on whole cell extracts of CCLP1 (**a**) and HuCCT-1 (**b**) cells, cultured in response to treatment with (rhPEDF) or without (CTR) 1000 ng/mL of rhPEDF for 24 h; GAPDH was used as a loading control. Quantification from three independent immunoblots by densitometry analysis is shown (for details see Section 2); ** *p* < 0.01 (*t*-test).

As previously reported, PEDF inhibits breast cancer cell migration and invasion by downregulating p-ERK and p-AKT signaling pathways [16]. Conversely, in nasopharyngeal carcinoma (NPC) PEDF expression suppresses NPC cell migration through the LRP6/GSK3β/β-catenin axis [27]. Therefore, we investigated which of these signaling pathways is involved in the rhPEDF-mediated inhibition of the iCCA cell motility. Using Western blotting, we analyzed the levels of p-ERK1/2, p-Akt and β-catenin. The results revealed that rhPEDF significantly decreased the phosphorylation of ERK1/2 in CCLP1 (Figure 5a) and HuCCT-1 (Figure 5b) cells. In contrast, no changes were observed in AKT phosphorylation nor β-catenin expression in either cell line.

## 4. Discussion

In the iCCA, PEDF along with THBS1 and THBS2 are expressed and released in the TME, where they play a role in the tumor progression. These factors hinder the neoangiogenic program, thereby promoting lymphangiogenesis and facilitating cancer cell dissemination through the lymphatic system [20]. While THBS1 and THBS2 also directly promote iCCA cell proliferation, adhesion and motility [21], in the present research we demonstrated that PEDF exhibits a tumor suppressor activity on iCCA cell lines by inhibiting their migration and invasion.

Identified in 1987, PEDF was initially recognized as a neurotrophic factor with a potent angioinhibitory effect. In recent years, however, this protein has emerged as one of the most promising anticancer agents acting directly on tumor cells. Its mechanism of action involves the inhibition of cell proliferation, activating different signal transduction pathways driven by its ability to act as a ligand for several receptors [9,10]. In prostate cancer, for instance, the binding of PEDF to PEDF-receptor/phospholipase A2 at the plasma membrane leads to the upregulation of Peroxisome Proliferator-Activated Receptor Gamma (PPARγ). This process, in turn, suppresses the nuclear factor-κB (NF-κB)-mediated transcriptional activation, thereby reducing the production of interleukin 8 (IL-8) and limiting the proliferation of prostate cancer cells [28]. Beyond the PPARγ/NF-κB/IL-8 axis, PEDF has been shown to bind the catalytic β-subunit of F1-ATP synthase present on the surface of various tumor cell lines inhibiting ATP production and decreasing the viability of both endothelial and tumor cell [29,30]. Furthermore, a recent study demonstrated that PEDF is implicated in suppressing autophagy through the downregulation of AMPK-ULK1 signaling. Specifically, PEDF inhibits the expression and activation of AMPK, triggering the inactivation of autophagy marker ULK1. This mechanism results in the inhibition of autophagy, and consequently, the suppression of non-small cell lung cancer cell proliferation [31]. Since we did not observe any changes in cell proliferation or viability, it is likely that these signaling pathways are not activated by exogenous PEDF on the iCCA cells.

Beyond its inhibitory effect on angiogenesis, the antimetastatic activity of PEDF is also mediated through the suppression of cancer cell motility [16,27,32,33,34,35]. In the context of iCCA, we demonstrated that exogenous PEDF impacts cancer cell motility by reducing migratory and invasive capabilities of both mesenchymal and epithelial cell lines. It is well established that cancer cell motility is suppressed by signaling pathways involved in the downregulation of promigratory genes or in inhibiting the remodeling of cytoskeletal and focal adhesions [36,37]. Among these pathways, the p-ERK and p-AKT signaling in breast cancer [16], the LRP6/GSK3β/β-catenin axis in NPC [27], and the cytosolic fatty-acid-binding protein 7 (FABP7)/PPARγ axis in malignant gliomas have been reported [38]. In this study, we observed a non-statistically significant decrease in p-AKT/AKT, exclusively in the CCLP1 cells treated with rhPEDF. In MDA-MB-231 breast cancer cells, PEDF decreases the overall abundance of insulin receptor substrate 1 (IRS1), which is known to limit the downstream p-AKT pathway and, consequently, the cancer cell survival and proliferation [39]. The discrepancy may be related to the differences in cell types resulting in a less pronounced decrease in the IRS1 levels. On the other hand, we reported that rhPEDF reduces the phosphorylation of ERK1/2 both in the CCLP1 and HuCCT-1 cell lines, suggesting the MAPK/ERK signaling pathway may mediate the PEDF-induced inhibition of the iCCA cell motility. ERK1 and ERK2 are largely known as the downstream components of a signaling pathway induced by growth factors and mitogen signals transduced by the RAS GTPases [40]. Although studies on ERK1/2 have primarily focused on their role in controlling cell proliferation, survival, growth and differentiation, increasing evidence highlights their involvement in regulating cellular responses such as metabolism and migration [41]. Recently, several reports have revealed how inhibition of the ERK1/2 signaling pathway significantly suppresses iCCA cell motility induced by various factors such as activation of the calcium-sensing receptor [42], the fibroblast growth factor receptor 2 [43], the rho-related GTP-binding protein RhoC [44], and the aldehyde dehydrogenase 3B2 [45]. Similar to the breast cancer [16], our study highlights PEDF as a new upstream signal capable of suppressing the p-ERK1/2 signaling pathway thereby counteracting iCCA progression.

We also found that PEDF inhibits cell adhesion of the epithelial HuCCT-1 cells, but it does not affect the adhesive properties of mesenchymal-like CCLP1 cells. These findings align with previous studies on the effects of the exogenous PEDF on cell attachment in epithelial-like or mesenchymal-like cancer cell lines. For instance, the administration of rhPEDF to two lung cancer epithelial-like cell lines (A549 and SK-MES1) significantly reduced tumor cell adhesion to the ECM (Matrigel) [46]. In contrast, treatment with PEDF on JJ012 cells, a mesenchymal-like chondrosarcoma cell line, enhanced their adhesion to the type-I collagen [47]. Therefore, since mesenchymal cells downregulate certain epithelial integrins while activating the expression of others [48], it is conceivable that the differing responses in cell attachment to the rhPEDF treatment may be due to the distinct integrin and adhesion molecules profiles between epithelial and mesenchymal cells. In fact, in a report comparing the expression profile of adhesion molecules in iCCA cells, it has been demonstrated that the CCLP1 and HuCCT-1 differ in the CD13 and CD90 expressions [22]. Particularly, CCLP1 are CD90^+^ CD13^−^ cells, while the HuCCT-1 shows a CD90^−^ CD13^+^ profile, thus suggesting that the two cell lines may be primed by different adhesion mechanisms. Moreover, since the transmembrane molecule CD13 is expressed on endothelial cells and has been shown to mediate cell adhesion [49], it is conceivable that the limited effect of PEDF on CCLP1 cells may be further related to the peculiar expression profile of adhesion molecules on the iCCA mesenchymal cell surface.

PEDF is not expressed by the iCCA cells, whereas THBS1 and THBS2 are expressed and released in the iCCA TME by both tumor cells and CAFs. As a result, thrombospondins exert a direct autocrine and paracrine prooncogenic role on tumor cells [21], while PEDF may only partially act as a tumor suppressor factor in this context. Notably, CAFs secrete a wide array of pro-migratory signals, such as periostin [50], THBS1 and THBS2 [21], osteopontin [51], and stromal cell-derived factor 1 [52] which may outweigh the oncosuppressive effects of PEDF on the iCCA cells.

## 5. Conclusions

This report presents the first evidence that rhPEDF, by directly binding to mesenchymal and epithelial iCCA cells, counteracts their migration and invasive capacities. We also demonstrated that treatment with rhPEDF can downregulate ERK1/2 phosphorylation levels, shedding light on the molecular mechanism underlying the paracrine action of PEDF on the iCCA cells. This study enhances our understanding of the multifaceted roles of PEDF in iCCA. Future research will focus on identifying the iCCA cell surface receptor targeted by PEDF. At present, in addition to the aforementioned F1-ATP synthase and PEDF-receptor/phospholipase A2, five other receptors, including laminin receptor (LR), lipoprotein receptor-related protein, plexin domain-containing 1 (PLXDC1), plexin domain-containing 2 (PLXDC2), and vascular endothelial growth factor receptor 2 (VEGFR2), have been reported to be high-affinity receptors for PEDF [4,53]. By retrieving data from the Human Protein Atlas, all these proteins are expressed in iCCA tissues with moderate to strong immunostaining intensities [54]. Identifying which of these receptors is engaged by PEDF could lead to the development of specific therapeutic strategies to effectively inhibit cancer cell migration, invasion, and adhesion, which are important determinants of the high aggressiveness of iCCA. To date, one of the main research efforts in counteracting the iCCA metastatic spread is focused on the development of therapeutic approaches that combine targeted therapies, immunotherapy and chemotherapy. For example, the combined use of bevacizumab with chemotherapy or with anti-PD-1/PD-L1 drugs may lead to a synergistic response in reducing metastasis and improving the overall survival rates of iCCA patients [55,56]. In the last years, exogenous PEDF has been introduced as part of a combined therapeutic approach to enhance both the efficacy of chemotherapies and the action of immune checkpoint inhibitors [57,58]. Thus, the combined approach of chemotherapies and immunotherapies with PEDF could help overcome treatment resistance and limit iCCA cell dissemination, offering promising new therapeutic options for the iCCA patients.

## Data Availability

Data are contained within the article and Supplementary Materials.

## Supplementary Materials

The following supporting information can be downloaded at: https://www.mdpi.com/article/10.3390/biology14020155/s1, Figure S1: Western blotting from Figure 2b,d and Figure 5a,b. Table S1: Primary antibodies used for Western Blot analysis in this study.
