# Supplementary material for: Pigment Epithelium-Derived Factor Inhibits Cell Motility and p-ERK1/2 Signaling in Intrahepatic Cholangiocarcinoma Cell Lines
Source: Biology (Basel). 2025 Feb 3;14(2):155. doi: 10.3390/biology14020155 (PMC11851717; doi:10.3390/biology14020155)
Supplement: Supplementary file 1 [file biology-14-00155-s001.zip › biology-3409334-supplementary.pdf]

Table S1. Primary Antibodies for Western Blot analysis

	Description	Tested Applications	Species Reactivity	Immunogen	Clonality	Dilution
anti-PEDF (Abcam, #14993)	Rabbit polyclonal antibody targeting Pigment Epithelium-Derived Factor (PEDF), a 50 kDa secreted glycoprotein.	WB, IF, IHC	Mouse, Human	Fusion protein (His-Tag) corresponding to Human PEDF. Corresponding to full length human PEDF expressed in baby hamster kidney cells.	Polyclonal	1:500
anti-PARP (Cell Signaling, #9532)	Monoclonal antibody specific for PARP-1, recognizing both the full-length form and the 89 kDa fragment produced by caspase cleavage at Asp214.	WB, IP, IF-IC, FC-FP	Human, Mouse, Rat, Monkey	Synthetic peptide corresponding to residues surrounding Gly623 of human PARP-1.	Monoclonal	1:1000
anti-PCNA (Cell Signaling, #13110)	Monoclonal antibody specific for PCNA, a nuclear protein involved in DNA replication.	WB, IP, IHC-P, IF-F, IF-IC, FC-FP	Human, Mouse, Rat, Monkey	Synthetic peptide corresponding to residues near the carboxy terminus of human PCNA protein.	Monoclonal	1:1000
anti-AKT (Cell Signaling, # 4691)	Monoclonal antibody specific for AKT, a serine/threonine kinase involved in various cellular processes.	WB, IP, IHC-P, IF-IC, FC-FP	Human, Mouse, Rat, Monkey, Drosophila Melanogaster	Synthetic peptide corresponding to residues in the carboxy-terminal sequence of mouse Akt.	Monoclonal	1:1000
anti-pAKT (Cell Signaling, # 4060)	Monoclonal antibody specific for AKT phosphorylated at Ser473, indicative of protein activation.	WB, W-S, IP, IHC-P, IF-IC, FC-FP	Human, Mouse, Rat, Monkey, Hamster, Zebrafish, Bovine, Drosophila Melanogaster	Synthetic peptide corresponding to residues surrounding phosphorylated Ser473 of human AKT.	Monoclonal	1:1000
anti-ERK1/2 (Cell Signaling, # 4695)	Monoclonal antibody specific for ERK1/2, kinases involved in cellular signal transduction.	WB, IP, IHC-P, IF-IC, FC-FP	Human, Mouse, Rat, Monkey, Hamster, Drosophila Melanogaster, Zebrafish, Bovine, Dog, Pig	Synthetic peptide corresponding to residues surrounding Thr202/Tyr204 of human ERK1.	Monoclonal	1:1000
anti-pERK1/2 (Cell Signaling, # 9101)	Polyclonal antibody specific for phosphorylated ERK1/2 at Thr202/Tyr204, indicative of protein activation.	WB, IP, IF-IC, FC-FP	Human, Mouse, Rat, Hamster, Monkey, Mink, Drosophila, Zebrafish, Bovine.	Synthetic peptide corresponding to residues surrounding Thr202/Tyr204 of phosphorylated human ERK1/2.	Polyclonal	1:1000
anti-β-catenin (BD Biosciences, #610153)	Monoclonal antibody specific for β-Catenin, a 92 kDa protein that binds to the cytoplasmic tail of E-Cadherin, playing a crucial role in cell adhesion.	WB, IF, IP, IHC	Human, Mouse, Rat, Dog, Chicken.	Mouse β-Catenin amino acids 571-781.	Monoclonal	1:500
anti-Actin (Santa Cruz Biotechnology, #sc-1615)	Polyclonal antibody targeting the C-terminus of Actin, suitable for detecting a broad range of Actin isoforms.	WB, IP, IF, FC-FP, ELISA	Mouse, Rat, Human, Xenopus laevis, Zebrafish, Caenorhabditis elegans.	Peptide mapping at the C-terminus of human Actin.	Polyclonal	1:1000
anti-GAPDH (Santa Cruz Biotechnology, #sc20357)	Monoclonal antibody specific for GAPDH (Glyceraldehyde-3-Phosphate Dehydrogenase), a 37 kDa enzyme involved in glycolysis.	WB, IF, IP, ELISA, IHC	Human, Mouse, Rat.	Peptide mapping within an internal region of GAPDH of human origin.	Monoclonal	1:1000

Fig 1b PEDF

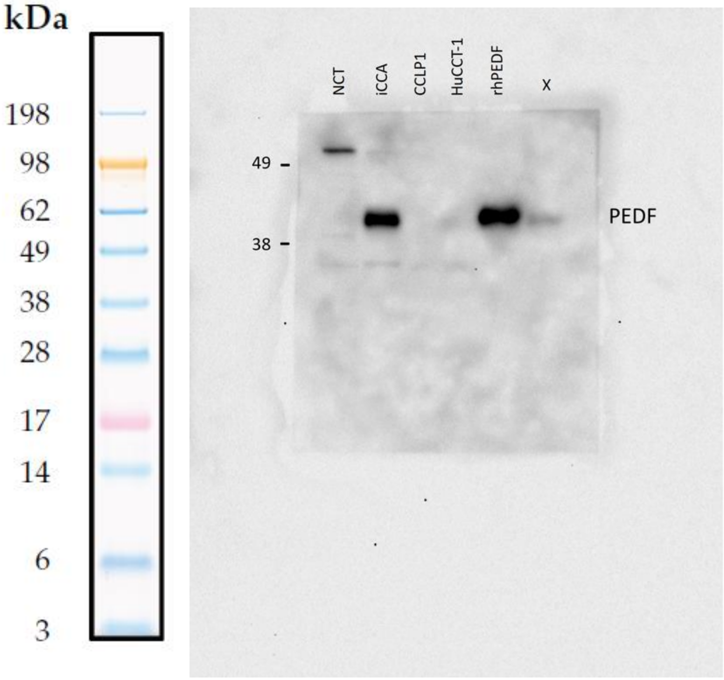

Fig 1b Ponceau S

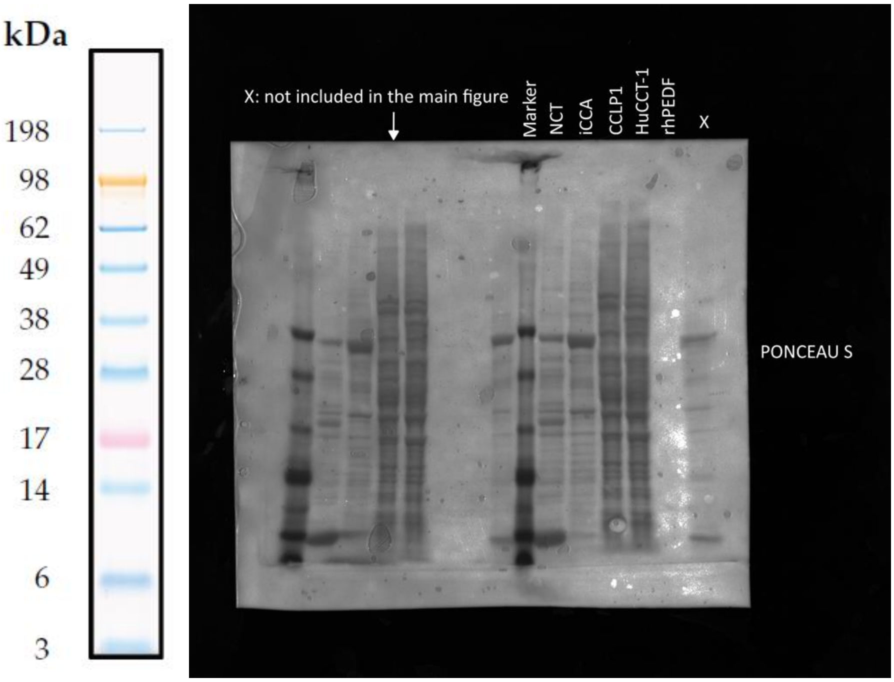

Fig 2b -d lanes 1 PARP-1

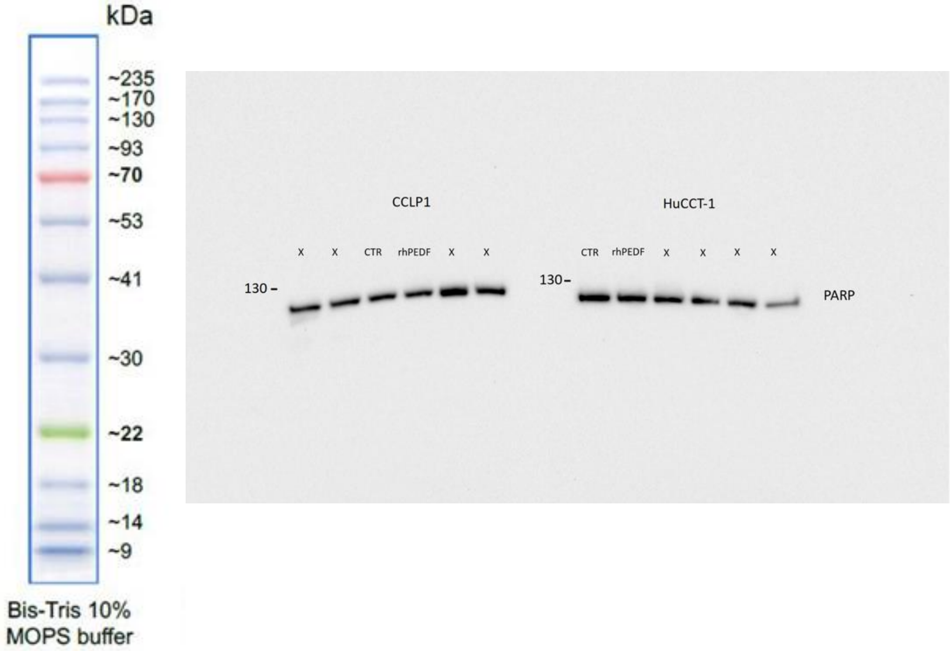

Fig 2b -d lanes 2 PCNA

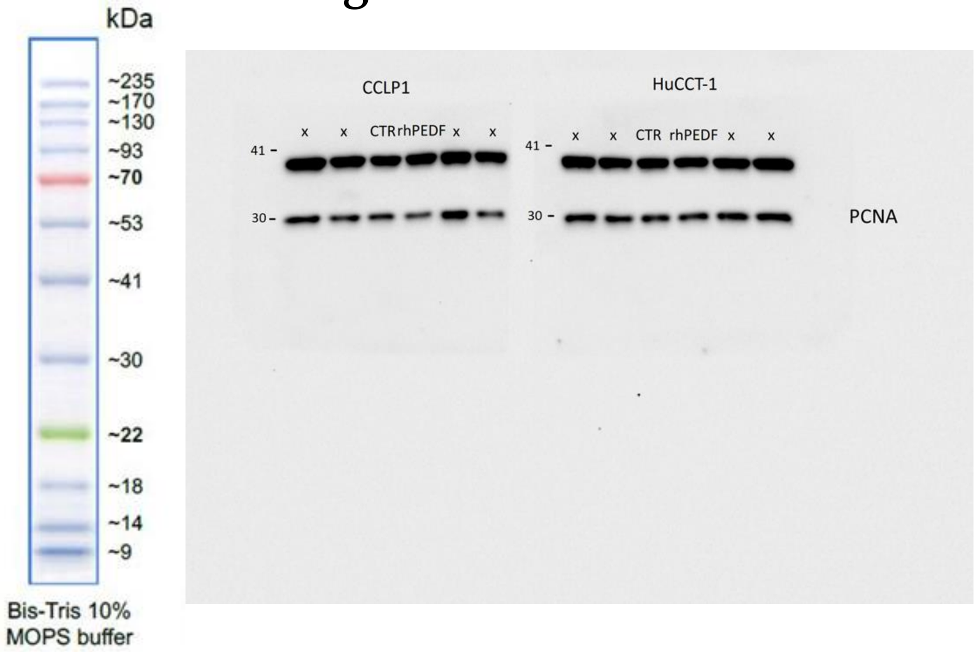

Fig 2b -d lanes 3 Actin

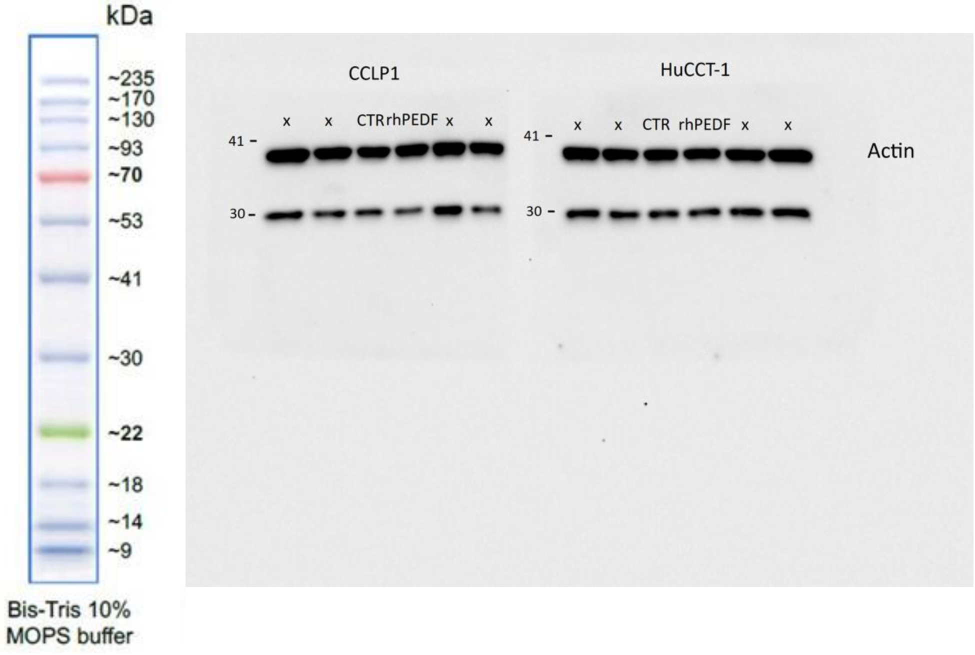

Fig 5a lanes 2 and 4. AKT and ERK1/2

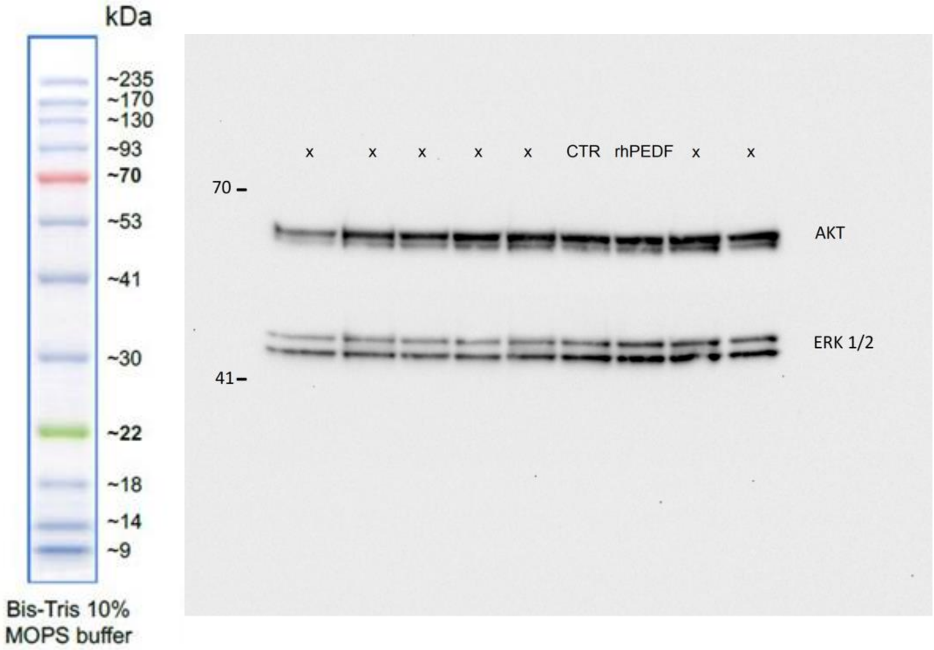

Fig 5a lanes 1. p-AKT

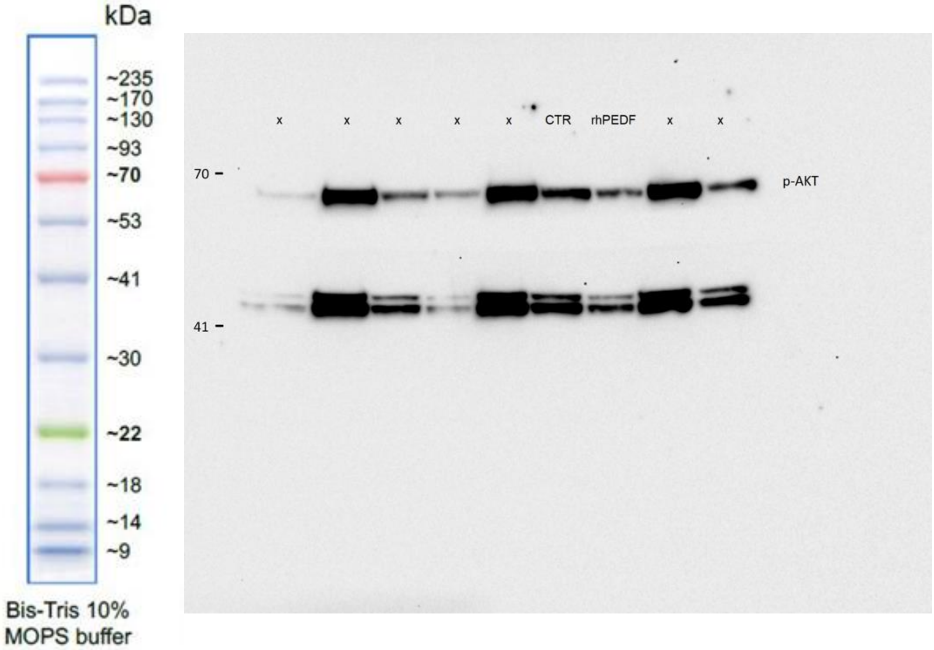

Fig 5a lane 3. p- ERK1/2

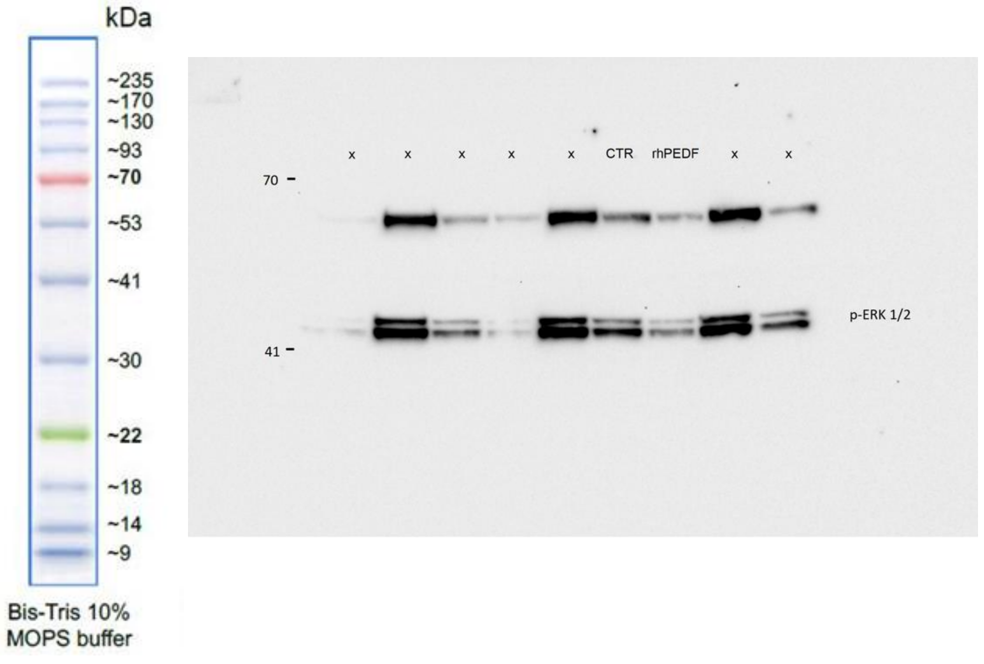

Fig 5a lane 5. β- catenin

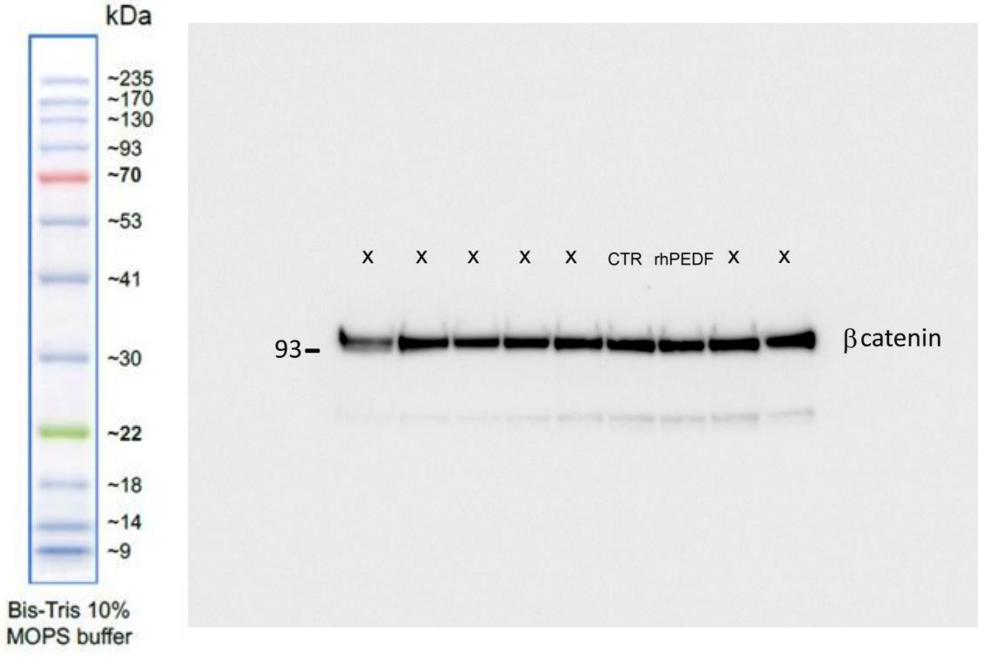

Fig 5a lanes 3. p - ERK1/2

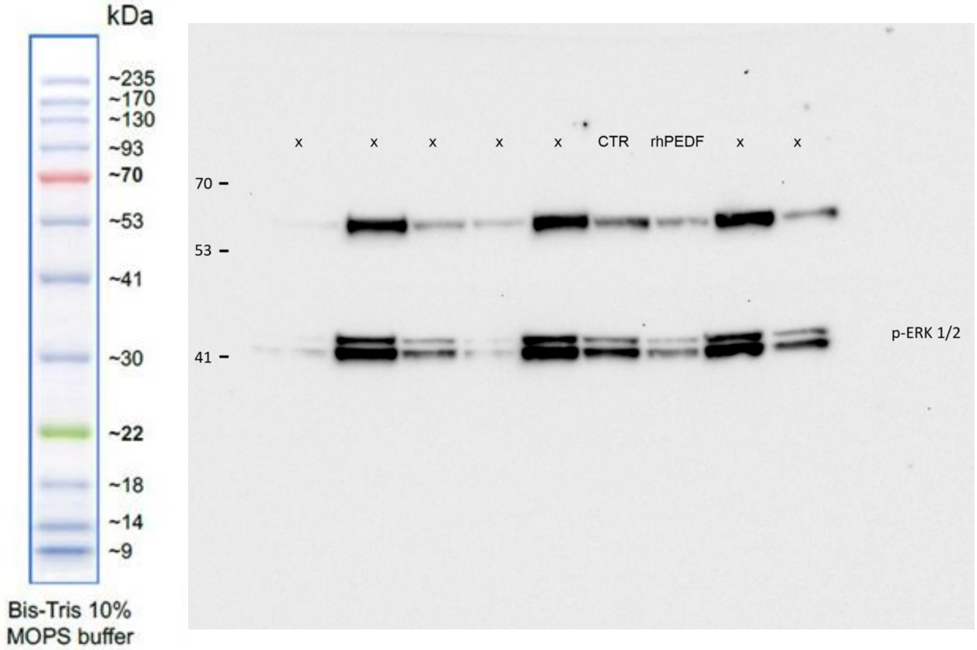

Fig 5a lanes 6. GAPDH

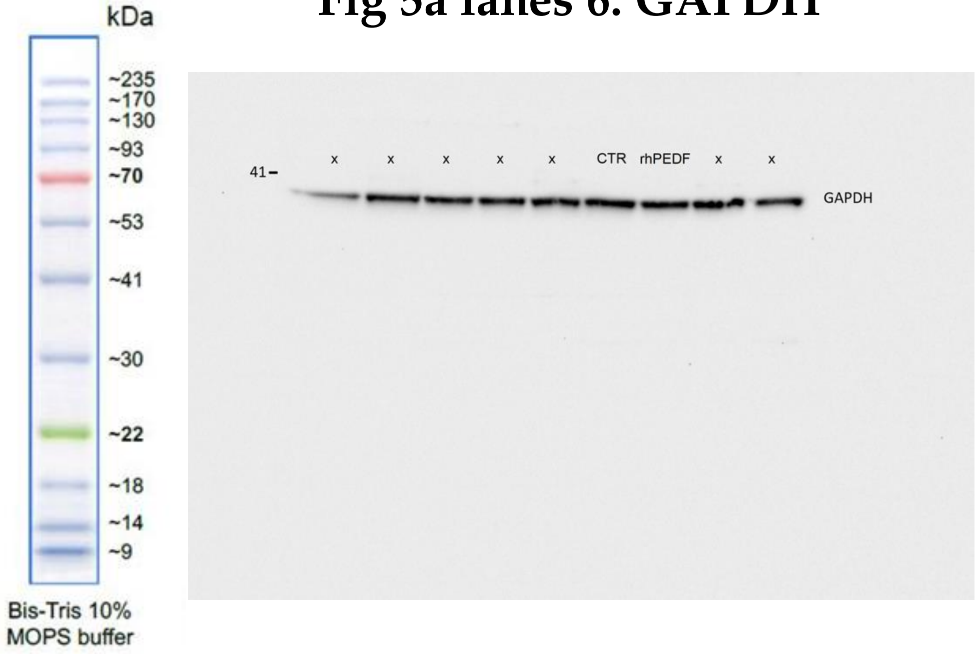

Fig 5b lanes 1. p - AKT

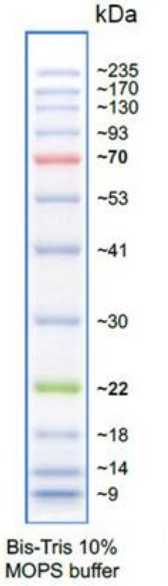

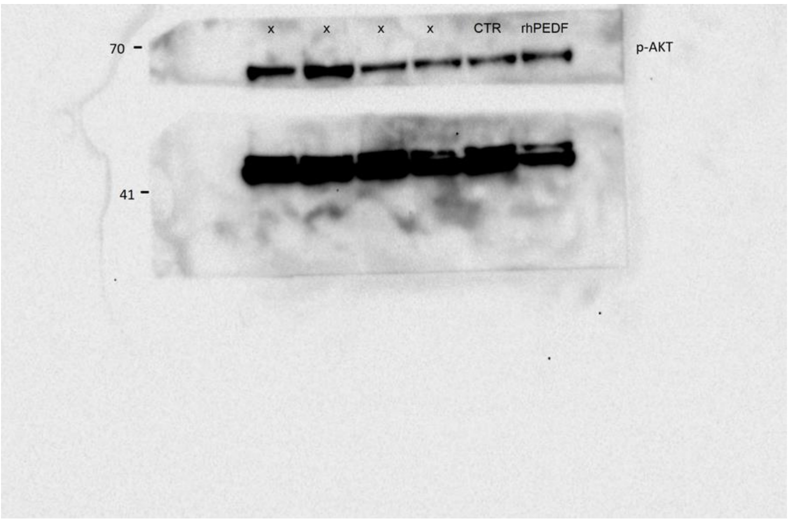

Fig 5b lanes 2,4,5. AKT, ERK1/2, β- catenin

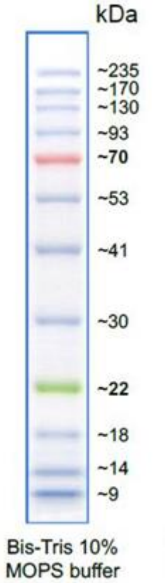

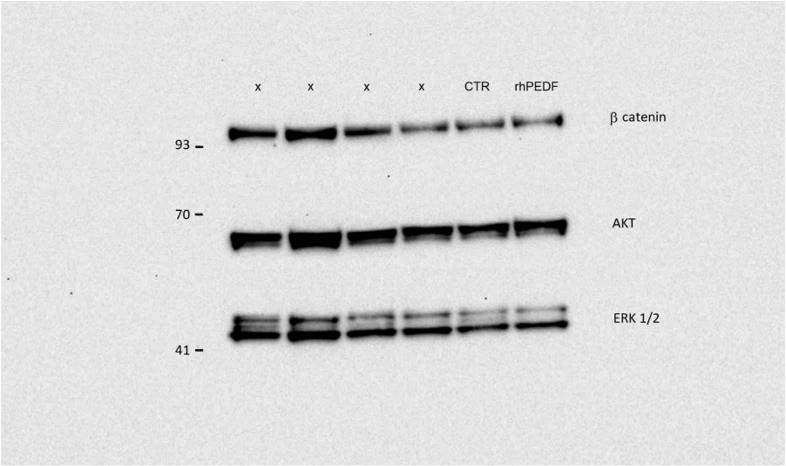

Fig 5b lane 3. p-ERK1/2

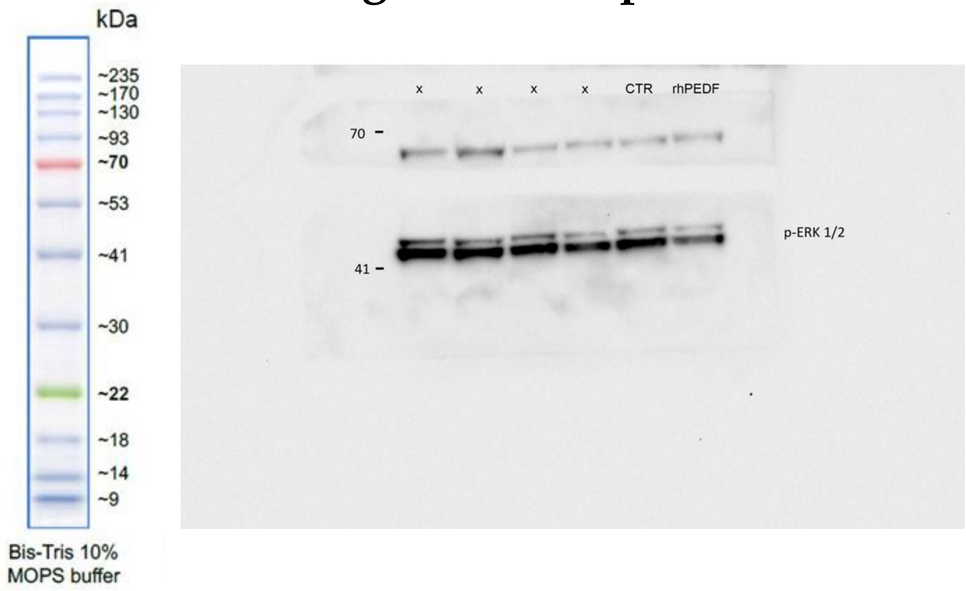

Fig 5b lane 6. GAPDH

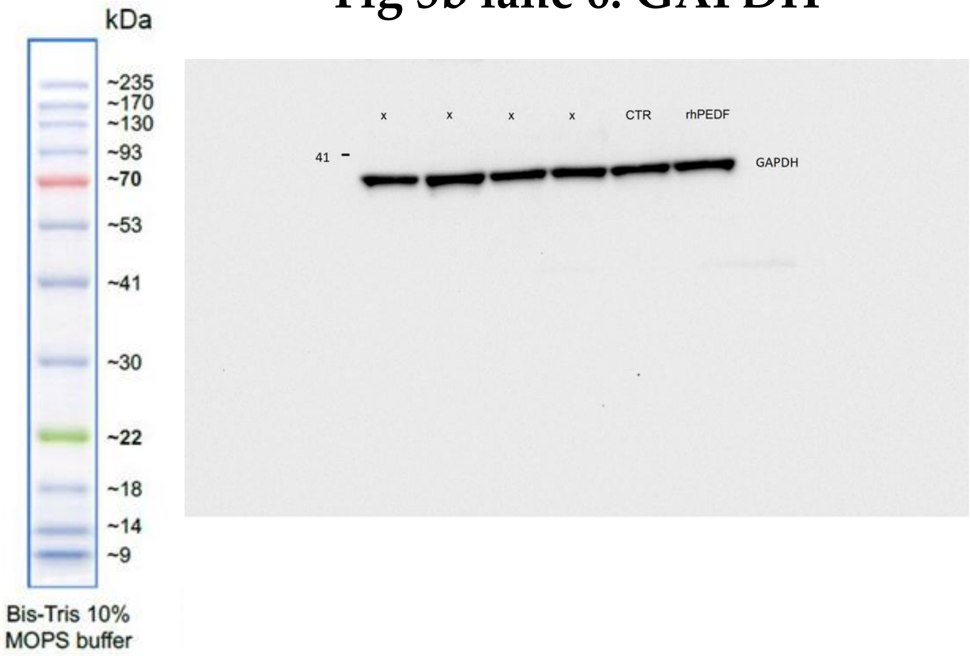

Figure S1: Western blotting from Figure 2b,d and Figure 5a,b.
